# HPV prevalence and risk of pre-cancer and cancer in regular immigrants in Italy: results from HPV DNA test-based screening pilot programs

**DOI:** 10.1186/s13027-015-0009-x

**Published:** 2015-05-07

**Authors:** Cinzia Campari, Chiara Fedato, Alessio Petrelli, Manuel Zorzi, Carla Cogo, Adele Caprioglio, Federica Gallo, Livia Giordano, Serena Domenighini, Luigi Pasquale, Sonia Prandi, Marco Zappa, Paolo Giorgi Rossi, the GISCi Migrant Working Group

**Affiliations:** Staff Programmazione e Controllo, AUSL, Reggio Emilia, Italy; IRCCS-Arcispedale Santa Maria Nuova, Reggio Emilia, Italy; Coordinamento regionale screening oncologici, Regione Veneto, Venezia, Italy; INMP Istituto Nazionale per la promozione della salute delle popolazioni Migranti ed il contrasto delle malattie della Povertà, Rome, Italy; Registro Tumori del Veneto, Regione Veneto, Padova, Italy; Unità di Epidemiologia - CPO Piemonte, Torino, Italy; ASL Valle Camonica Sebino, Breno, BS Italy; Patologia IRCCS-Arcispedale Santa Maria Nuova, Reggio Emilia, Italy; ISPO – Istituto per lo Studio e la Prevenzione Oncologica, Florence, Italy; Servizio Interaziendale di Epidemiologia, AUSL, Reggio Emilia, Italy

## Abstract

**ᅟ:**

Immigrants from low- and medium-income countries have a higher risk of cervical cancer due both to barriers in access to screening and to higher human papillomavirus (HPV) prevalence.

In the near future many screening programmes in industrialised countries will replace Pap test with HPV as primary test. In order to plan future interventions, it is essential to understand how the HPV screening performs in immigrant women.

**Methods:**

We conducted a survey on the main performance indicators from some of the HPV DNA-based pilot programmes in Italy, comparing regular immigrant women, identified as women resident in Italy who were born abroad, with women who were born in Italy. All the programmes applied the same protocol, with HPV as stand-alone test starting for women of 25 or 35 to 64 years of age. Cytology triage is performed for positive women; those ASC-US or more severe are referred directly to colposcopy; negative women are referred to repeat HPV after one year.

**Results:**

Overall, 162,829 women were invited, of whom 22,814 were born abroad. Participation was higher for Italy-born than born abroad (52.2% vs. 43.6%), particularly for women over 45 years. HPV positivity rate was higher in immigrants: 7.8% vs. 6.1%, age-adjusted Relative Risk (age-adj RR) 1.18, 95% confidence interval (95% CI) 1.13-1.22. The proportion of women with positive cytology triage was similar in the two groups (42%). Cervical Intraepithelial Neoplasia (CIN) grade 2 or more severe detection rate was higher for born abroad (age-adj RR 1.65, 95% CI 1.45-1.89). The difference was stronger when considering only CIN3 or more severe (age-adj RR 2.29, 95% CI 1.90-2.75). Both HPV positivity and CIN2 or more severe detection rate had a different age curve in born abroad compared with Italy-born: in the former, the risk was almost flat, while in the latter it declined rapidly with age.

**Conclusion:**

Compliance with HPV screening is lower for migrant women, who are affected by higher HPV positivity and CIN3 cancer detection rates.

## Introduction

Persistent infection with oncogenic HPV types is the necessary, but not sufficient, cause of cervical cancer [1,2]. Wide differences in cervical cancer incidence and mortality have been observed around the world, with more than 85% of invasive cancers occurring in low- and middle-income countries [3]. The differences in cancer incidence are mostly related to the diffusion of Pap test use and screening programmes [4-6].

Several studies have shown that migrant women from low- and medium- income countries have higher risk of cervical cancer [7-11] and usually maintain the same cervical cancer risk as that of their countries of origin for several years [12-15]. Both of the main determinants of cervical cancer can be related to the differential risk in immigrant women: many countries of origin have high prevalence of HPV infection [16,17]; Pap test coverage in migrant women is lower than in native women because the diffusion of screening in their Countries of origin is very low [17] and because there could be several barriers to screening access in the host country [5,18-22].

Migration in Italy is a relatively recent phenomenon: in 1991, foreigners resident in Italy were less than 1% of the total population while in 2011 they were 7.5%, with some cities over 16%. Most of immigrant women in Italy are in the target age of cervical cancer screening [23,24].

Screening with HPV DNA as primary test has been demonstrated to be more effective in preventing cancer [25]. Many international guidelines are now introducing HPV test as primary screening test [26-30].

Screening programmes in Italy cover the vast majority of the resident population, without any difference with regard to citizenship [31]. In 2007, the first pilot programme using HPV DNA as primary screening test was implemented [32], followed by several other experiences in 2009–10 [33-36]. All the screening programmes, including HPV DNA pilot programmes, systematically collect information about invitations, test results, colposcopy and histology, and participate in a national survey [37,38]. In 2012, the survey included a focus on immigrant women.

### Objective

In this paper we compare the screening results in 7 HPV-based pilot screening programmes in foreign-born vs. Italy-born women.

## Materials and methods

### Setting

In Italy, according to the European Commission recommendations (2003) [39], the Regional Health Systems are committed to organize screening programmes that actively invite the target female population. The national guidelines recommend a Pap test every three years for women aged 25–64 years. All women resident must be invited and all screening tests, ascertainments and treatments are free of charge.

Since 2007, after the publication of the results of three large randomised trials on HPV DNA as primary test [40-42], some Local Health Authorities started HPV DNA-based pilot programmes. All the programmes adopted the same protocol: [43] HPV DNA as primary test, followed by triage with cytology: HPV positive (HPV+) and cytology positive (cyto+) prompted immediate colposcopy, while HPV+ cytology negative (cyto-) prompted one-year follow up with HPV. Some pilot programmes adopted this strategy for women age 25–64 years, others for women age 35 to 64 only. The interval for HPV negative (HPV-) women was 3 years for all women recruited until 2012. Women receive the test results by letter if negative; they are contacted by phone in case of positivity (and also by letter, in some programmes only if there is no answer; in other programmes, always) to make an appointment for colposcopy. In some programmes women who are HPV positive and cytology negative are contacted only by letter while in some other programmes they are contacted by phone. In any case women are actively contacted to communicate the results of the screening tests.

Some of the projects were randomised pragmatic trials; the others were intervention studies with historical or geographical controls. The pilot programmes tested feasibility, organizational impact, acceptability (for operators and women), compliance with invitation and follow-up protocols, performance of cytology triage and, finally, they collected field information for economic and budget impact analysis.

All the projects were approved by the local Ethics Committees or were established by law by the regional health authority.

The Osservatorio Nazionale Screening, supported by the Centro per la Prevenzione Oncologica (CPO), Piedmont, annually collects aggregated data from all the Italian screening programmes, whose results are published in an annual national report [37,38].

The survey methods were developed by the GISCi (Gruppo Italiano per lo Screening Cervicale) in 1999 [44], and are consistent with the minimum dataset of indicators of the European guidelines [45].

From 2012, the survey collected information from the HPV DNA-based screening.

### The migrant survey

In 2012, the GISCi promoted the collection of information on immigrant women inside the national survey. The country of birth was used to identify the migrant status. Only two programmes (Reggio Emilia and Torino) classified the countries as low- and medium-income or other (i.e., high income), according to the classification of “high migration pressure countries” of the Italian Ministry of Health [46].

Data were collected with the same form as the official survey and were disaggregated by age and migration status, while they did not distinguish women at first vs. subsequent screening episodes. Furthermore, we requested data for the three-year period 2009–11.

### HPV DNA molecular analyses

HPV test was performed on liquid based cytology samples or on samples specific to HPV test. In the latter case, a smear was obtained and fixed before taking the sample for molecular testing.

All the programmes used Hybrid Capture 2 (Qiagen), on both liquid based cytology (PreservCyt) or on Standard Transport Medium (STM). For programmes using two samples (cytology and STM) the denaturation and hybridization was directly performed on the sampling medium. For programmes using liquid based cytology, 4 ml of PreservCyt were transferred for the conversion according to the manufacturer’s instructions. HPV results were classified as positive or negative for high-risk types. The types included were 16, 18, 31, 33, 35, 39, 45, 51, 52, 56, 58, 59, and 68.

### Cytology

In the programmes using conventional smears, only the slides of HPV+ women were stained and interpreted. In the case of liquid based cytology, the slide was prepared from residual material after HPV testing only when HPV+. Cytology results were classified according to TBS 2001. All the programmes referred women with ASC-US or more severe cytology to colposcopy. Women with an unsatisfactory cytology were referred to colposcopy in some programmes, while cytology was repeated on a new sample in others.

### Analysis

We present the set of the indicators routinely used to evaluate the performance of screening programmes, stratified by country of birth (Italy vs. other countries). For two programmes, migrants were further stratified in women from low/medium income and from high income countries. The indicators included participation (crude and adjusted – i.e., accounting for the undelivered mails), HPV positivity, cytology triage results, recall rate to colposcopy, compliance with colposcopy, detection rate for cervical intraepithelial neoplasia grade 2 (CIN2), CIN grade 3 (including Adenocarcinoma in situ, CIN3), and for cancer at baseline (i.e., not including one-year follow up for HPV+ cytology- women). Except for participation and compliance to colposcopy, all indicators were age-standardised using the Italy-born screened women as standard population. For participation, cytology results, and detection rate, we calculated age-adjusted relative risks (age-adj RR) with 95% confidence intervals (95% CI).

## Results

Seven of the 19 pilot projects active in Italy in 2012 participated in the survey. The duration of the study period ranged from one to three years; the survey was restricted to screening results of women invited between 1 January 2009 and 31 December 2011. Overall, the pilot programmes invited 162,829 women, of whom 22,814 were immigrant (Table 1).

**Table 1:** **Characteristics of the participating centres: target population, proportion of foreigners, study period, and available information for immigration status**

				**Period**			**Information about immigration status**		
**Centre**	**Target population for HPV screening (Years)**	**Invited population (N)**	**Proportion of born abroad (%)**	**2009**	**2010**	**2011**	**Place of birth**	**Citizenship**	**Identifying high migration pressure countries**
Torino	35-64	41,440	18.1	+	+	+	+	+	+
Alta Padovana	25-64	22,826	15.4			+	+	+	
Padova	25-64	15,459	15.4			+	+	+	
Este	25-64	31,963	8.9		+	+	+	+	
Rovigo	25-64	15,534	11.4			+	+	+	
Adria	25-64	7,464	8.5			+	+		
Reggio Emilia	35-64	17,048	18.3		+	+	+	+	+
Valcamonica	25-64	11,095	9.5			+	+		

In the programmes that distinguished women from low/medium income countries vs. those from high income countries, the latter accounted for a small proportion of all the migrants (9.4%).

**Figure 1:**
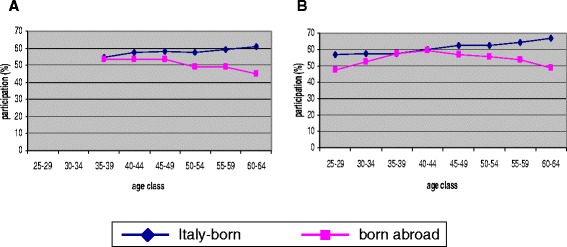
Participation in HPV-based cervical cancer screening by age and place of birth. **A**. Torino and Reggio Emilia, target age 35–64. **B**. Veneto and Valcamonica, target age 25–64.

Women born abroad invited for screening were on average younger than Italians (mean age 43.3 vs. 46.7 years, respectively). The participation was lower in born abroad than in Italy-born women (43.6% vs. 52.2%), though the difference was noticeable only in women aged ≥ 40 years (Figure 1).

The overall participation rate in women from high-income countries was lower than the average of women born abroad (44% vs 47% and 61% vs 65% in Turin and Reggio Emilia, respectively).

**Figure 2:**
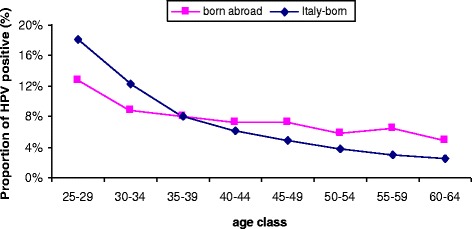
Proportion of women HPV positive (Hybrid Capture II, high-risk types) by age and place of birth.

The overall age-adjusted HPV positivity rate was higher in born abroad than in Italy-born women (age-adj RR 1.18, 95% CI 1.13-1.22), with a different shape by age (Figure 2): the curve for Italians decreased sharply with age, while the migrants showed a modest decrease between ages 25 and 40 and a second peak after menopause. As a result, young women born abroad had a lower HPV prevalence than did young Italy-born women. Women born in high income countries showed a prevalence curve very similar to Italians.

Among HPV+ women, the prevalence of cyto + was similar in migrants and Italians, resulting in higher referral to both colposcopy and one-year follow up for migrants. Women born abroad had a higher proportion of high-grade cytology (H-SIL, ASC-H, and cancer) than did Italians (10.1% vs. 5.9% of the HPV+ women, p = 0.0001) (Table 2).

**Table 2:** **Main data and principal performance indicators of seven Italian pilot programmes adopting HPV as primary screening test followed by cytology triage, by country of birth**

					**Relative risk immigrants vs. Italians**			
	**Italy-born**		**Born abroad**		**Crude RR**	**95% CI**	**Age adjusted RR**	**95% CI**
	**N**	(%)*	**N**	(%)*				
Invited Population (N)	140,015		22,814					
Undelivered letters	1,305	0.9	1196	5.2				
Participants	73,023	52.2	9941	43.6	0.84	(0.82-0.85)		
Participation excluding undelivered letters (%)		52.6		46.0	0.87	(0.86-0.89)		
HPV tests performed	72,176		9680					
HPV positive	4,421	6.1	751	7.8	1.27	(1.18-1.36)	1.18	(1.13-1.22)
cytological triage*								
*Negative*	*1,925*	*48.4*	*326*	*49.2*				
*Inadequate*	*180*	*4.5*	*24*	*3.6*				
*ASC-US*	*444*	*11.2*	*66*	*10.0*				
*AGC*	*17*	*0.4*	*5*	*0.8*				
*L-SIL*	*1,173*	*29.5*	*174*	*26.3*				
*ASC-H*	*74*	*1.9*	*22*	*3.3*				
*H-SIL*	*158*	*4.0*	*42*	*6.3*				
*Cancer*	*3*	*0.1*	*3*	*0.5*				
*Total ASC-US+ (% of HPV positive)*	*1,869*	*47.0*	*312*	*47.1*	*0.98*	*(0.87-1.07)*	*0.95*	*(0.90-0.99)*
Referred to colposcopy	1,968	2.7	324	3.3	1.23	(1.09-1.38)	1.22	(1.09-1.37)
Compliance to colposcopy	1,840	93.5	297	91.7	0.98	(0.95-1.02)		
Detection rate for CIN3+ (‰ screened women)	163	2.3	50	5.2	2.29	(1.67-3.14)	2.29	(1.90-2.75)
Detection rate for CIN2+ (‰ screened women)	342	4.7	79	8.2	1.72	(1.35-2.20)	1.65	(1.45-1.89)
*CIN2*	*179*	*52.3*	*29*	*36.7*				
*CIN3 + adeno* in situ	*153*	*44.7*	*45*	*56.7*				
*Invasive Cancer*	*10*	*5.6*	*5*	*17.2*	*3.73*	*(1.27-10.90)*	*3.9*	*(1.33-11.41)*
Positive predictive value of colposcopy referral		18.6		26.6	1.43	(1.16-1.77)	1.38	(1.12-1.71)

*cytology triage results were missing for 447 and 89 women born in Italy and abroad, respectively.

Compliance to colposcopy referral was high in both groups (91.7% and 93.5% for immigrants and Italians, respectively).

**Figure 3:**
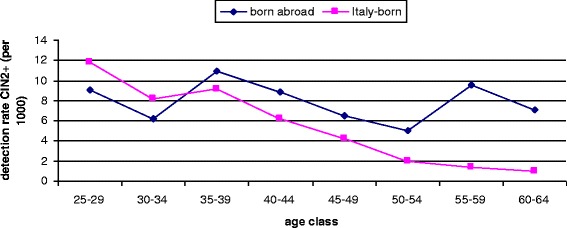
Detection rate of CIN2+ by age and place of birth.

The detection rate of CIN2+ was higher for women born abroad than for Italians (8.2‰ vs. 4.7‰). The difference was entirely due to an excess of CIN3+ (age-adj RR 2.29, 95% CI 1.90-2.75), while a negligible difference was observed for CIN2 (age-adj RR 1.07, 95% CI 0.88-1.31) (Table 2). The detection rate was different also by age: the risk was lower for women born abroad than for Italians below age 30, while at older ages the risk decreased in Italians but remained stable in women born abroad, resulting in an excess of CIN2+ in the latter in women older than 40 years 40 (Figure 3). Women from high income countries showed an overall age-adjusted detection rate lower than that of Italians.

Five of the 15 cancers identified was found in women born abroad, who accounted for only 12% of the screened population. In fact, the cancer detection rate was about four times higher in women born abroad than in Italians (age-adj RR 3.7, 95% CI 1.3-11.4).

## Discussion

This is the first study reporting the results of several population-based screening programmes that adopted HPV DNA as primary screening stratified for migrant and native population in an industrialized country. In Italy, HPV DNA-based screening was implemented quite early: the first pilot programme started in 2007, [32] and in January 2013 the Ministry of Health adopted the HTA Italian report, [47] thus allowing the Public Health Service to introduce HPV DNA as primary screening.

### Participation

Several of the HPV DNA test pilot programmes reported a slightly higher participation than that of Pap test-based programmes [32-36]. However, this increase is unlikely to reduce the gap in participation between Italians and immigrants. In fact, our survey detected a difference in participation between Italians and immigrants, both from high income and from low/medium income countries, similar or even higher than that observed in Pap test-based programmes [48].

Nevertheless, population-based screening programmes are effective interventions in reducing inequality in the access to evidence-based prevention [49-51]. In Italy, the difference in Pap test coverage between Italians and immigrants is smaller in the regions where well-organized screening programmes are present, compared to those regions where screening programmes cannot regularly invite all the population and the access to Pap test is mainly spontaneous [52]. This effect occurs even when the participation is slightly lower for immigrants than for Italians, but given the much lower uptake of opportunistic screening by immigrants, organised programmes contribute to reducing inequality, which should be the case with HPV DNA-based screening as well.

### Prevalence of HPV infections and high-grade lesions

The prevalence of HPV infection was higher in immigrants than in Italians. Also, the curve of prevalence by age was different in the two groups: the results in Italian women confirmed the prevalence curve already observed in previous population-based studies [53-55], with a constant decline with age, which was steep until age 40 and flatter from ages 45 to 65. The prevalence in immigrants, instead, decreased slightly from ages 25 to 35 and then remained almost flat.

The curve in immigrants is similar to that of many low/medium income countries such as sub-Saharan Africa, central Asia, and Latin America [56], but not to some of other countries most represented among immigrants resident in Italy (e.g. Mediterranean Africa, the Middle East, and Indian sub-continent [17,56]. The foreign population in Italy is extremely heterogeneous in terms of country of origin: the first 5 countries (Romania, Albania, Morocco, China, and the Ukraine) account for only 50% of the foreign population [24]. Furthermore, the different populations are not evenly distributed by age [24]. Thus, the overall prevalence curve probably reflects the distribution by age of some communities with a very high prevalence in their countries of origin, such as sub-Saharan Africa and Eastern Europe, and of other communities with a low HPV prevalence, such as Mediterranean Africa and Indian sub-continent. The latter immigrants are younger, while women from Eastern Europe, an area with a very high prevalence, tend to be older.

It is worth noting that the overall proportion of cyto + among HPV+ women is very similar in Italians and immigrants, while the proportion of high-grade lesions is higher in immigrants. A higher proportion of women with high-grade cytology is consistent with a higher detection rate of CIN2+ and particularly of CIN3 in immigrants than in Italians. A ratio between high-grade and low-grade lesions in favour of high grade is probably linked to a lower uptake of screening among immigrants in the preceding years, both in their countries of origin and in Italy. This means that long-lasting and more severe lesions are detected. Indeed, a higher detection rate may be the consequence of both higher HPV prevalence and lower screening uptake in the preceding years.

All of the observed differences between immigrants and Italians suggest that the immigrant populations maintain the prevalence of their countries of origin for several years, particularly when migration is a relatively recent phenomenon (as it is in Italy). Many other studies have also observed this phenomenon when looking at HPV prevalence [16,57], cytological abnormalities [18,19,48], and pre-cancer or cancer risk [5,7-15].

### Limits and strengths

The country of birth, which we used as a proxy of the migration status, could misclassify women, thereby including many Italian women among the migrants. Nevertheless, even citizenship, unanimously considered a better proxy of migrant status in women aged 25–64, can misclassify a significant number of women who are married to Italians but who migrated to Italy relatively recently. Another Italian study compared cervical cancer screening indicators obtained using the country of birth or citizenship and found only minor differences [48]. We decided to use the country of birth because this information was more uniformly collected by the programmes and it was probably the most reliable.

Furthermore, only few programmes could distinguish women from low/medium income from those from high-income countries. All the analyses confirmed that, in Italy now, calculating the indicators for all the foreigners provides figures that substantially depict the health status of immigrants.

Differences between native and immigrant women tend to decrease with time after the arrival in the host country [15]. In the case of cervical cancer risk, it would be interesting to observe the time needed to reduce the gap in screening participation and in HPV prevalence. Unfortunately we do not have information on the length of stay in Italy.

On the other hand, the characteristics of immigrants from high-income countries were substantially comparable to those of Italians, with the exception of participation.

Another limit of this study is that we could not identify the exact country of origin. HPV prevalence differs in different areas and it would be important to distinguish between countries with high vs. low prevalence, in order to determine whether the prevalence of communities resident in Italy reflects the country of origin. Analyses by specific communities are necessary to better understand the epidemiology of HPV and cervical cancer in immigrants and to target public health interventions.

Furthermore, we could not distinguish between women at their first screening round and those who had already had a previous Pap test. On the other hand, it must be noted that almost all the women included in this survey were at their first HPV screening test.

Finally, screening programmes actively invite only women that are resident, or at least registered, in their catchment area, while irregular migrants and asylum seekers, who are surely more vulnerable than are regular residents, can only access screening spontaneously. Therefore we have information only on regular immigrants, registered as such. While citizens from outside of the European Union (i.e. all the regular immigrants but not tourists or those with a short-term or a study visa) must be registered residents, the same is not true for European Union citizens. However, since residents can obtain tax reductions, reduced fees for electricity, gas, water supplies, and so on, not being resident has many disadvantages. Thus, persons staying for a long time usually are registered as resident.

## Conclusions

Immigrant women have a higher prevalence of HPV, a worse ratio of high-grade vs. low-grade lesions, and consequently a higher risk of cervical pre-cancer and cancer than do Italians. The prevalence of HPV seems to reflect that of their countries of origin. HPV DNA-based programmes are not likely to reduce the gap in participation in screening programmes between immigrants and Italians.
